# Identification of markers that distinguish adipose tissue and glucose and insulin metabolism using a multi-modal machine learning approach

**DOI:** 10.1038/s41598-021-95688-y

**Published:** 2021-08-23

**Authors:** Josefin Henninger, Björn Eliasson, Ulf Smith, Aidin Rawshani

**Affiliations:** 1 The Lundberg Laboratory for Diabetes Research, Department of Molecular and Clinical Medicine, The Sahlgrenska Academy at the University of Gothenburg, 413 45 Gothenburg, Sweden; 2 Department of Molecular and Clinical Medicine, Institute of Medicine, University of Gothenburg, Gothenburg, Sweden; 3 Wallenberg Laboratory for Cardiovascular and Metabolic Research, Institute of Medicine, University of Gothenburg, Gothenburg, Sweden

**Keywords:** Computational biology and bioinformatics, Molecular biology, Physiology, Biomarkers, Endocrinology, Medical research, Molecular medicine, Risk factors

## Abstract

The study of metabolomics has improved our knowledge of the biology behind type 2 diabetes and its related metabolic physiology. We aimed to investigate markers of adipose tissue morphology, as well as insulin and glucose metabolism in 53 non-obese male individuals. The participants underwent extensive clinical, biochemical and magnetic resonance imaging phenotyping, and we also investigated non-targeted serum metabolites. We used a multi-modal machine learning approach to evaluate which serum metabolomic compounds predicted markers of glucose and insulin metabolism, adipose tissue morphology and distribution. Fasting glucose was associated with metabolites of intracellular insulin action and beta-cell dysfunction, namely cysteine-s-sulphate and n-acetylgarginine, whereas fasting insulin was predicted by myristoleoylcarnitine, propionylcarnitine and other metabolites of beta-oxidation of fatty acids. OGTT-glucose levels at 30 min were predicted by 7-Hoca, a microbiota derived metabolite, as well as eugenol, a fatty acid. Both insulin clamp and HOMA-IR were predicted by metabolites involved in beta-oxidation of fatty acids and biodegradation of triacylglycerol, namely tartrate and 3-phosphoglycerate, as well as pyruvate, xanthine and liver fat. OGTT glucose area under curve (AUC) and OGTT insulin AUC, was associated with bile acid metabolites, subcutaneous adipocyte cell size, liver fat and fatty chain acids and derivates, such as isovalerylcarnitine. Finally, subcutaneous adipocyte size was associated with long chain fatty acids, markers of sphingolipid metabolism, increasing liver fat and dopamine-sulfate 1. Ectopic liver fat was predicted by methylmalonate, adipocyte cell size, glutathione derived metabolites and fatty chain acids. Ectopic heart fat was predicted visceral fat, gamma-glutamyl tyrosine and 2-acetamidophenol sulfate. Adipocyte cell size, age, alpha-tocopherol and blood pressure were associated with visceral fat. We identified several biomarkers associated with adipose tissue pathophysiology and insulin and glucose metabolism using a multi-modal machine learning approach. Our approach demonstrated the relative importance of serum metabolites and they outperformed traditional clinical and biochemical variables for most endpoints.

## Introduction

Obesity is a multifactorial and heterogeneous disorder that is generally associated with metabolic alterations such as insulin resistance and type 2 diabetes, as well as a major risk factor for cardiovascular- morbidity and mortality. Adipose tissue constitutes of subcutaneous-, visceral- and peripheral “ectopic” fat depots, but functional variations in adipose tissue depots mediate discrepancies in metabolic and atherosclerotic risk. Failure of adipocyte growth and differentiation results in acquired lipodystrophy and pathologic fat accumulation. Upon excess caloric intake, energy is preferably stored in subcutaneous adipose tissue, which initially expands by hyperplastic growth, but in predisposed individuals, the subcutaneous adipose tissue fails to do so and instead exhibits cell dysfunction associated with adipocyte hypertrophy, mild inflammation and fibrotic remodeling. Adipose tissue dysfunction is considered a hallmark of type 2 diabetes and a major contributor to the development of insulin resistance, which in addition to β-cell dysfunction and impaired insulin secretion, forms the cornerstones of type 2 diabetes biology^1–3^.

Understanding of biological mechanisms underpinning these conditions is constantly evolving and the addition of metabolomics has resulted in improved diagnosis and prognosis of metabolic disorder, increased our understanding of adipocyte biology and insulin- and glucose metabolism^4,5^. Previous research indicates that metabolites reflecting glycolytic and tricarboxylic acid cycle (TCA) intermediates, branched-chain and aromatic amino acids, and long-chain fatty acids are associated to metabolic disorders^6–8^.

Recently, our research group presented data that certain metabolites correlated to genetic predisposition to type 2 diabetes, impaired glucose tolerance, insulin resistance, adipocyte hypertrophy, and to ectopic fat accumulation, in healthy and lean study participants with- and without heredity for type 2 diabetes^9^.

In this study, using adipose tissue biopsies and magnetic resonance spectroscopy, we set out to investigate candidate markers for morphological characterization of subcutaneous adipose tissue and dysfunction, along with markers for visceral adipose tissue and lipid accumulation in ectopic depots. In addition, we investigated markers of insulin- and glucometabolism based on clinical characteristics, biochemical variables, non-targeted metabolites and magnetic resonance spectroscopy data. For this end, we constructed multi-modal predictive machine learning models to manage this high-dimensional dataset, with emphasis on untargeted serum metabolomics.

## Methods

### Ethics statement

All subjects received oral and written information and gave informed consent to participate. The study protocol was approved by the local Ethical Committees at the Sahlgrenska Academy at the University of Gothenburg (approvals 384-12 and T803-13). The study was performed in agreement with the Declaration of Helsinki.

### Study population

We recruited 53 subjects via newspaper advertisements and through earlier studies performed at the laboratory. Inclusion criteria were male sex and general good health. The data collection of biochemical variables, radiological examinations and clinical variables have been described previously^9^.

### Clinical variables

Lifestyle factors, as well as number of relatives diagnosed with type 2 diabetes mellitus, were evaluated through a questionnaire filled out in the laboratory.

Body weight and height, and waist and hip circumferences were recorded. We used bioelectrical impedance (single frequency, 50 kHz; Animeter, HTS, Odense, Denmark) to determine the proportions of body fat and lean body mass. Blood pressure was measured with a mercury sphygmomanometer in a sitting position after a 5 min rest.

### Biochemical variables

After 12 h of fasting all subjects underwent an OGTT (75 g glucose orally) to assess glucose tolerance status. Samples for measurement of plasma glucose and serum insulin were drawn after 0, 30, 60 and 120 min. Using fasting plasma insulin and fasting plasma glucose from the OGTT, we calculated a HOMA-IR index using the formula HOMA-IR = (fasting plasma glucose x fasting plasma insulin)/22.5^10^. M and M/I following euglycemic clamps were used to validate the HOMA-IR.

To determine the first and second phases of insulin secretion, an intravenous glucose tolerance test (IVGTT) was performed after another overnight fast. A bolus of glucose (300 mg/kg in a 50% solution) was given within 30 s into the antecubital vein. Samples for the measurement of plasma glucose and insulin (arterialised venous blood) were drawn at − 5, 0, 2, 4, 6, 8, 10, 20, 30, 40, 50 and 60 min. Using the trapezoidal method, we calculated the acute and the late insulin responses, i.e. incremental area under the insulin curve, (AIR, 0–10 min; LIR, 10–60 min). These parameters were not included in prediction models due to co-linearity with oral glucose and insulin tolerance tests. Preliminary prediction models suggested that OGTT derived predictors had greater relative importance, compared to IVGTT predictors.

All subjects underwent a hyperinsulinemic euglycaemic clamp (insulin infusion: 240 pmol m^−2^ min^−1^ for 120 min), after another 12 h fast, to asses insulin sensitivity^11^. Whole blood glucose was clamped at 5.0 mmol/l for the next 120 min by infusion of 20% glucose at various rates according to glucose measurements performed at 5 min intervals (YSI, Yellow Springs Instrument Company, OH). The M value (insulin sensitivity) was calculated as the mean glucose infusion rate during the last 30 min of the clamp adjusted for total body weight. M/I was calculated as the M-value corrected for steady-state insulin concentrations.

Plasma glucose was measured using standard laboratory methods (Department of Chemistry, Sahlgrenska University Hospital, Gothenburg, Sweden). Plasma insulin was measured at the University of Tübingen, Germany, by micro-particle enzyme immunoassay (Abbott Laboratories, Tokyo, Japan).

From each subject we obtained a subcutaneous abdominal adipose tissue biopsy to assess subcutaneous adipose tissue cell size. The biopsies (approximately 1–200 mg) were obtained with a needle aspiration technique, and further processed to evaluate adipose tissue cell size as previously stated^9,12^. All metabolites were measured in serum after a 12 h fast.

### Radiological variables

Magnetic resonance imaging (MRI) was used to assess the amount of intra-abdominal and subcutaneous fat. Localised ^1^H-magnetic resonance spectroscopy was used to assess liver fat and heart lipids. MRI and MRS were performed using a 1.5 T MR-system (Intera/Achieva, software release 3.2) using the vendor’s 16 channel SENSE XI Torso coil (Philips Medical Systems, Best, The Netherlands). The software used included a research package enabling navigator triggered MRS and a field map based B_0_-shimming. MRI images were evaluated at the level between the 4th and 5th lumbar vertebrae using T1 weighted axial images. MRI data was processed using an in-house developed segmentation program written in MatLab (MATLAB R2014b, The MathWorks Inc., USA). The surface of intra-abdominal and subcutaneous adipose tissue was quantified. Bone, muscle, lean tissue as well as inter-muscular fat were excluded. The fat fractions are reported as ratios to total body volume. MRS liver data and MRS cardiac data were processed using the jMRUI software. Magnetic resonance methods have been further reported in previous scientific works^9^.

### Statistical analysis

Baseline characteristics for clinical-, biochemical-, metabolic- and imaging markers are presented as mean ± SD, for all study participants and cluster subgroups identified with k-means clustering method (Table 1).

**Table 1:** Baseline characteristics for all study participants including 3 unique clusters that were identified through k-means clustering method.

Characteristics	Entire cohort	Cluster 1	Cluster 2	Cluster 3	*p*-value
N	53	14	20	19	
Age (years)	42.26 (8.23)	38.36 (8.26)	40.26 (7.24)	46.79 (6.86)	0.004
Diastolic blood pressure (mmHg)	80.79 (9.99)	79.71 (9.78)	76.89 (8.89)	84.42 (9.69)	0.055
Systolic blood pressure (mmHg)	127.30 (12.24)	126.43 (11.27)	122.47 (13.30)	131.42 (9.89)	0.068
Body mass index kg/m^2^	25.57 (3.52)	23.23 (1.53)	25.94 (3.58)	27.20 (3.55)	0.003
Body weight (kg)	83.69 (13.13)	76.29 (9.57)	85.62 (12.69)	87.98 (13.37)	0.025
Waist circumference (cm)	90.30 (9.62)	84.21 (5.89)	91.24 (8.84)	93.47 (10.36)	0.014
Serum creatinine (micromol/L)	88.42 (10.49)	91.00 (10.93)	86.32 (10.87)	89.95 (8.34)	0.361
Waist to hip ratio	0.89 (0.06)	0.85 (0.05)	0.88 (0.05)	0.90 (0.06)	0.032
Resting hear trate (amount/min)	56.92 (7.64)	54.50 (5.00)	56.00 (7.33)	58.79 (9.00)	0.251
Serum TSH (mIE/L)	1.98 (1.01)	2.07 (1.03)	1.75 (1.02)	2.15 (1.03)	0.469
Serum sodium (mmol/L)	140.68 (1.76)	140.79 (2.22)	140.79 (1.55)	140.16 (1.42)	0.449
Serum white blood cell count 10^9/L)	4.73 (1.06)	4.15 (0.64)	4.78 (1.16)	5.01 (1.12)	0.065
Serum potassium (mmol/L)	4.29 (0.23)	4.25 (0.23)	4.31 (0.26)	4.32 (0.21)	0.673
Serum hemoglobin (g/L)	147.91 (10.25)	145.79 (9.27)	146.37 (10.16)	149.68 (10.71)	0.474
Serum free T4 (nmol/L)	15.75 (2.06)	16.36 (2.10)	15.58 (1.89)	15.26 (1.91)	0.283
Serum bilirubin (micromol/L)	11.85 (6.84)	14.39 (7.59)	11.39 (6.57)	11.42 (6.87)	0.397
Liver transaminases ratio	1.00 (0.33)	1.04 (0.31)	0.94 (0.24)	1.01 (0.42)	0.648
Serum ASAT (microkat/L)	0.43 (0.12)	0.40 (0.10)	0.42 (0.13)	0.47 (0.13)	0.258
Serum ALAT (microkat/L)	0.47 (0.19)	0.39 (0.10)	0.49 (0.22)	0.52 (0.20)	0.157
Serum alkaline phosphatase (microkat/L)	1.00 (0.29)	1.02 (0.28)	1.04 (0.38)	0.95 (0.17)	0.564
Fasting plasma glucose (mmol/L)	4.93 (0.41)	4.79 (0.48)	4.99 (0.40)	5.01 (0.32)	0.244
Fasting serum insulin (pmol/L)	45.38 (22.26)	35.27 (14.00)	47.92 (26.85)	50.63 (21.16)	0.127
OGTT plasma glucose after 30 min (mmol/L)	8.25 (1.61)	7.95 (1.85)	8.75 (1.81)	8.01 (1.20)	0.271
OGTT plasma glucose after 60 min (mmol/L)	7.71 (2.10)	7.33 (2.17)	7.74 (2.37)	7.96 (1.93)	0.706
OGTT plasma glucose after 120 min (mmol/L)	5.41 (1.73)	5.24 (1.76)	4.91 (1.58)	5.89 (1.99)	0.233
OGTT serum insulin after 30 min (pmol/L)	396.76 (210.49)	308.56 (135.92)	455.45 (246.05)	434.54 (204.49)	0.112
OGTT serum insulin after 60 min (pmol/L)	484.19 (369.15)	338.32 (204.27)	514.30 (412.33)	573.15 (405.19)	0.187
OGTT serum insulin after 120 min (pmol/L)	251.24 (205.36)	187.67 (170.92)	219.14 (159.64)	326.78 (258.87)	0.120
Area under the curve OGTT glucose	13.84 (2.87)	13.29 (3.26)	13.88 (3.04)	14.18 (2.71)	0.701
Area under the curve OGTT insulin	698.49 (426.07)	510.67 (239.34)	735.00 (459.08)	823.18 (466.23)	0.106
Glycated hemoglobin (mmol/mol)	32.92 (2.31)	33.24 (1.31)	32.79 (2.68)	33.11 (2.26)	0.830
MRS visceral fat	86.52 (47.11)	70.04 (44.15)	81.17 (37.71)	99.41 (57.04)	0.203
Subcutaneous fat	221.80 (88.76)	157.79 (64.42)	253.75 (92.21)	242.48 (77.49)	0.003
MRS Whole abdomen	590.45 (128.23)	509.80 (95.62)	613.79 (121.81)	626.78 (123.48)	0.014
HOMA-IR	10.82 (5.65)	8.75 (4.04)	12.08 (7.16)	11.26 (4.78)	0.236
Liver fat	61.18 (10.31)	58.65 (13.61)	60.90 (10.82)	63.57 (6.34)	0.400
Peripheral arterial insufficience (%)					0.343
0 = none	51 (96.2)	13 (92.9)	19 (100.0)	18 (94.7)	
1 = 1 first-degree relative	1 (1.9)	0 (0.0)	0 (0.0)	1 (5.3)	
5 = 1 SDR or 1 TDR	1 (1.9)	1 (7.1)	0 (0.0)	0 (0.0)	
Heredity for diabetes (%)					0.453
0 = none	21 (39.6)	5 (35.7)	10 (52.6)	7 (36.8)	
1 = 1 first-degree relative	12 (22.6)	5 (35.7)	2 (10.5)	5 (26.3)	
2 = 2 first-degree relative	4 (7.5)	1 (7.1)	1 (5.3)	2 (10.5)	
3 = 1 FDR and 1 SDR	1 (1.9)	0 (0.0)	1 (5.3)	0 (0.0)	
3 = 1 FDR and 1 SDR	6 (11.3)	0 (0.0)	2 (10.5)	4 (21.1)	
4 = 1 FDR or 1 SDR with type 1 diabetes	1 (1.9)	0 (0.0)	1 (5.3)	0 (0.0)	
5 = 1 SDR or 1 TDR	8 (15.1)	3 (21.4)	2 (10.5)	1 (5.3)	
Physical activity (%)					0.703
1 = Never	9 (17.0)	2 (14.3)	4 (21.1)	1 (5.3)	
2 = 1/week	4 (7.5)	0 (0.0)	2 (10.5)	2 (10.5)	
3 = 2–3 times/week	19 (35.8)	4 (28.6)	6 (31.6)	9 (47.4)	
4 = 4–6 times/week	14 (26.4)	5 (35.7)	4 (21.1)	5 (26.3)	
5 = Every day	7 (13.2)	3 (21.4)	3 (15.8)	2 (10.5)	
Insulin clamp ratio	0.02 (0.01)	0.02 (0.01)	0.02 (0.00)	0.02 (0.00)	0.026
MRS—Liver fat	3.40 (4.49)	1.20 (1.30)	3.32 (5.58)	4.96 (4.40)	0.060
Intensity of physical activity	35 (66.0)	11 (78.6)	13 (68.4)	10 (52.6)	0.284
Smoking habits	5 (9.4)	0 (0.0)	2 (10.5)	3 (15.8)	0.310
Adipocyte cell size (microm)	95.62 (11.64)	91.66 (13.53)	96.66 (10.08)	97.37 (12.22)	0.355
MRS—Heart fat	5.86 (2.86)	6.13 (3.69)	5.41 (2.14)	6.41 (3.00)	0.560

### Scaling of predictors in dataset

We construct extreme gradient boosting models to identify predictors for certain endpoints. These decision trees are generally considered invariant to monotonic transformations of features and node splits on one scale has a corresponding split on the transformed scale. However, extreme gradient boosting includes a linear booster and in the case of regularized regression, these models could be sensitive to feature scaling. Therefore, we have constructed both primary machine learning analyses based on Pareto scaled values for predictors and ancillary analyses of unscaled predictors. The ancillary analyses are presented in supplementary Appendix.

### Prediction models

Predictive machine-learning models were constructed with extreme gradient boosting, a decision-tree-based ensemble non-parametric algorithm that applies a gradient boosting framework. Our multi-modal and high-dimensional data necessitates a robust and validated predictive machine learning model to examine relative variable importance, i.e. predictive ability of a broad range of predictors.

Extreme gradient boosting applies parallelized implementation for sequential tree construction with tree pruning depending on negative loss criterion and splits up to the max depth, backwards tree pruning, defined through hyperparameter optimization, includes sparsity awareness and uses LASSO and Ridge regularization to prevent overfitting. Hyperparameter optimization was performed for each machine-learning model on the entire dataset, subsequent to automated grid search for number of trees, maximum depth of a tree, L2 regularization, learning rate, the fraction of observations, parameters to be randomly sampled for each tree and the minimum sum of weights of all observation required in a tree node. Finally, each optimized model was validated with repeated cross-validation, using 5 to 10 iterations for various models proved to be optimal and allowed for hyperparameter optimization to be based on the entire dataset. Moreover, in each prediction model, in parallel with our examination of optimal number of folds for the repeated cross-validation, we also scrutinized the pattern for feature importance to present a final model with maximum consistency in feature importance. For each outcome, five different machine learning models were constructed. We assessed feature importance on the entire dataset (henceforth referred to as the complete dataset), and four additional prediction models that included various data pre-processing techniques for dimension reduction of metabolomics data. For each outcome, features with highest relative importance from the five different prediction models were afterwards presented in a final figure. A graphical illustration is presented in the supplementary Appendix (Fig. S6), which demonstrates the model construction, optimization and validation for primary analyses. A similar modeling approach was performed for identical outcomes on the unscaled dataset and these results are presented in Figs. S3–S5.

### Feature extraction

In this study, we applied different dimensionality reduction techniques to non-targeted metabolomics parameters in order to reduce dimensions of feature space, whilst minimizing information loss. The non-targeted metabolomics data contains an excessive number of predictors for this dataset and there are presumably an abundance of metabolites that may not have any relationship with the endpoints being investigated. Principal component analysis (PCA) was performed to project scaled metabolomics data into lower dimensional space, reveal inherent data structure and provide a reduced dimensional representation of the original parameters. Principal component analysis was performed on the metabolomics separately and each machine-learning model included the first 20 principal components, which comprised of 75% cumulative variance.

In addition, we used a non-linear dimension reduction method called T-distributed stochastic neighboring embedding with an initial PCA step, perplexity at 10, theta 0.5 and 500 iterations, the metabolomics data was ultimately presented as three unique dimensions that were included in every prediction model. Moreover, exploratory factor analysis (EFA) is considered a data reduction technique and aims at explaining the relationship of many observed variables by a relatively small number of factors. The number of factors for EFA was decided using a simulated parallel analysis. We generated regression scores with 12 factors for EFA, using varimax rotation and minimum residual as factoring method.

Metabolomic data transformed with PCA, T-SNE and EFA were included in each gradient boosting model previous to automated grid search and hyperparameter optimization for the final model. Furthermore, we constructed two additional models that were based on recursive feature elimination with random forest and a complete dataset model that used all predictors (approximately 670 predictors). In the prediction models that demonstrated strong predictive ability for a parameter generated by means of dimension reduction techniques, we identified the unique predictors with peak scores in each dimension reduction model and included these predictors in the final linear regression model. In some instance, the predictions models based on the complete dataset or recursive feature elimination displayed similar metabolites as a model based on dimension reduction parameters. These metabolites were included once in the final linear regression model.

### Cluster analyses

In order to distinguish unique metabolic phenotypes with distinct differences in baseline characteristics or prediction modelling, we used k-means and hierarchical clustering. Model validation for k-means clustering was measured with the Elbow-, Silhouette- and Gap statistic model. Optimal number of *k* for cluster generation ranged between 2 and 3 clusters. ANOVA was performed for the metabolic markers of interest and baseline characteristics for individuals in the cluster groups are presented in Table 1. Results from k-means clustering were compared to hierarchical clustering. Supplementary Appendix displays the tanglegram results for hierarchical clustering, which was computed with the complete and Ward method, using Euclidean distance matrix.

### Linear regression models

Predictors with greatest relative importance identified through machine learning models were included in linear regression for assessment of effect size and significance level. Machine-learning models were used as a feature elimination method prior to feature selection for linear regression. The regression estimates and 95% confidence intervals are presented next to each machine learning model. The regression models were generated using log-transformed variables and standardized regression coefficients. Through linear regression, predictors with statistical significance were passed to identify variables of importance with linear regression.

### AUC for glucose and insulin metabolism

We applied the following trapezoid formula to assess area under the curve for glucose- and insulin levels after oral glucose tolerance test:$\user2{AUC~}\left( {\user2{Insulin}{ \setminus }\user2{Glucose}} \right) = \left( {\user2{Insulin}{ \setminus }\user2{Glucose}} \right)\left( {\user2{t}0} \right) + \left( {\left( {\user2{Insulin}{ \setminus }\user2{Glucose~}} \right)\user2{~}\left( {\user2{t}30} \right)\user2{x} \times 2} \right) + \left( {\left( {\user2{Insulin}{ \setminus }\user2{Glucose}} \right)\left( {\user2{t}60} \right) \times 3} \right) + \left( {\left( {\user2{Insulin}{ \setminus }\user2{Glucose}} \right)\left( {\user2{t}120} \right) \times \user2{~}2} \right)/4$.

### Imputation

We used missForest package in R to impute missing data for study participants, this package is based on the random forest algorithm. We analyzed distributions and means before and after imputation without observing virtually any differences. In general, the dataset had minor missing data. A *p*-value of less than 0.05 were considered to indicate statistical significance.

Calculations were performed in R (v 4.0.2) using the following machine learning libraries: XGBoost, Rtsne, Cluster, missForest, Caret, Psych, GPArotation, ggRandomForests, Party, GridExtra, mlr3, factoextra, Boruta, and Matrix.

## Results

### Study population

The study includes 53 men with a mean age of 42 ± 8 years. Initially, the study cohort was constructed to investigate adipose tissue morphology and metabolism in middle-aged, healthy, lean or mildly overweight non-diabetic individuals with heredity for type 2 diabetes, henceforth referred to as first-degree relatives (FDR), compared to individuals without heredity, henceforth referred to as control subjects (CTR). Almost half of the study participants (n = 22) had a known family history of type 2 diabetes. For all study participants, mean body mass index was 25 ± 3 kg/m^2^, mean fasting plasma glucose was 4.9 ± 0.4 mmol/L and mean fasting serum insulin was 45 ± 22 pmol/L. All study participants had normal liver function, systolic- and diastolic blood pressure and no ongoing pharmacological therapy. Baseline characteristics for k-means identified cluster groups are presented in Table 1. Mean values for age, body mass index, waist-circumference, MRS—subcutaneous fat, MRS—whole abdomen and insulin clamp ratio, differed among the three cluster groups.

### Insulin- and glucometabolic markers

**Figure 1:**
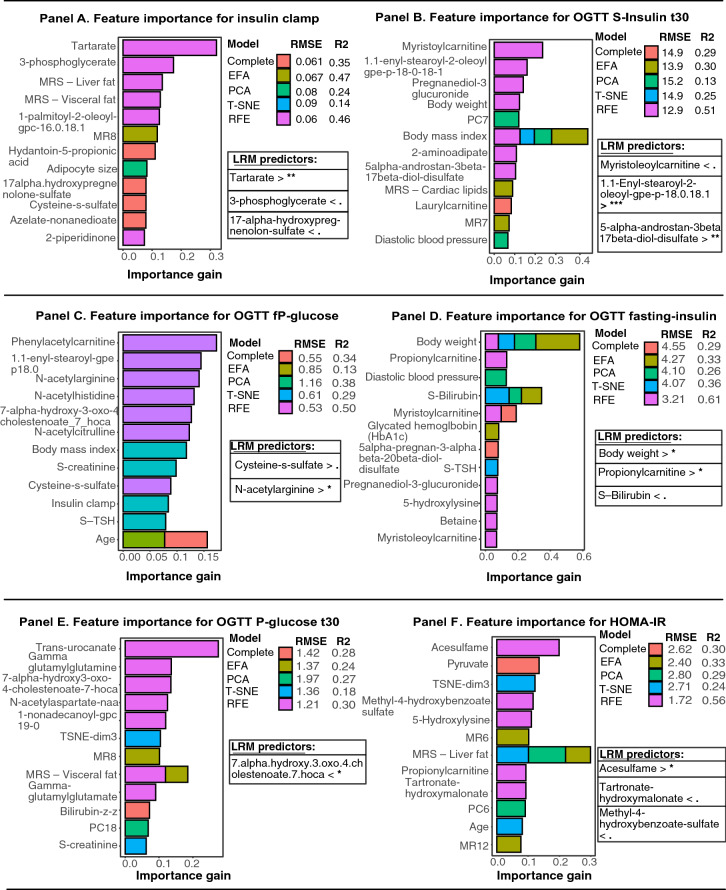
Feature importance for insulin- and glucometabolic markers based on extreme gradient boosting models and linear regression for the most important predictors. Panel (**A**) to Panel (**F**) displays relative importance for predictors generated by extreme gradient boosting models, using several pre-processing techniques for metabolomics data to further reduce number of predictors in the final machine-learning model. Model diagnostics (**RMSE**) and validation (**R**^**2**^) are presented next to each prediction model. For each outcome, the most important predictors identified through machine learning were included in linear regression models. All regression models were adjusted for age. Significance level are described as follows: *p-value < 0.05, **p-value < 0.005, ***p-value < 0.0005. *p*-values < 0.095. < denotes that lower levels for the predictor was associated with the target variable. > denotes that higher levels for the predictor was associated with the target variable.

Figure 1 (panel A–F) displays machine learning models for insulin- and glucometabolic markers along with linear regression models for the most important predictors identified through variable importance from predictive models. Each machine learning models treated metabolomics data differently.

### Hyperinsulinemic-euglycemic clamp

As shown in Fig. 1 Panel A, the strongest predictor of insulin clamp, in prediction models based on scaled values, was tartrate, 3-phosphoglycerate and fatty-chain acid metabolite, as compared to models with unscaled predictors, which shows that tartarate, 3-phoshpglycerate and MRS—liver fat, were the most important predictors (Supp Fig. S3 Panel A). As seen in Fig. 1 Panel A, the model with complete dataset did not generate predictors with strong predictability. The direction for standardized beta-coefficients are presented in Fig. 1 Panel A, linear regression for unstandardized beta-coefficients associated with insulin clamp was tartarate (βeta 1.25; 95% CI, 1.07 to 1.46), 3-phosphoglycerate (βeta 0.79; 95% CI, 0.68 to 0.92) and MRS—liver fat (βeta 0.94; 95% CI, 0.87 to 1.007) (Supp Fig. S3 Panel A).

### OGTT S-insulin after 30 min

In Fig. 1 Panel B, the prediction model based on exploratory factors (EFA) had the highest R^2^ (0.51) and lowest RMSE (12.9). Recursive feature elimination, T-SNE and PCA models displayed poor model diagnostics. Linear regression for the most important scaled predictors, revealed that myristoleoylcarnitine, enyl-stearoyl-2-oleoyl and 5-alpha-androstan-diol-sulfate, were associated with serum-insulin after 30 min, compared to the unscaled models, which identified body mass index, flavin-adenine dinucleotide-fad and 1.1 enyl palmitoyl-2-oleoyl-gpe, as statistically significant predictors (Fig. 1 Panel B and Supplementary Fig. S3 Panel B).

### OGTT fasting plasma-glucose

In Fig. 1 Panel C, following predictors displayed strong predictability for fasting-plasma glucose, n-acetylgarginine and cysteine-s-sulfate. All prediction models demonstrated relatively poor model diagnostics for the target variable. Linear regression, from both the scaled and unscaled predictions models, showed that Cysteine-s-sulfate and n-acetylgarginine, were important and significant predictors for this outcome (Fig. 1 Panel C and Supplementary Fig. S3 Panel C).

### OGTT fasting serum-insulin

Figure 1 Panel D shows the results for OGTT fasting insulin levels. The predictors propionylcarnitine, body weight and serum-bilirubin displayed strong predictability in several machine learning models, and both the scaled- and unscaled dataset. Linear regression demonstrated that body weight, propionylcarnitine and s-bilirubin were allmost statistically significant, (Supp Fig. S3 Panel D).

### OGTT plasma-glucose after 30 min

For the prediction model of plasma-glucose after 30 min, data pre-processing techniques demonstrated poor model diagnostics in both the scaled and unscaled dataset. Prediction models based on scaled predictors (Fig. 1 Panel E) suggest that *7-Hoca*, a microbiota -derived metabolite, was the most important predictor. Model diagnostics (R^2^ and RMSE-value), were greater in the unscaled models. The unscaled models identified, body weight, eugenol sulfate, S-ALAT and MRS—Liver fat were important predictors, however no predictor demonstrated statistical significance in the regression model, except eugenol sulfate, which was nearly significant (βeta 1.04; 95% CI, 0.98 to 1.09) (Supp Fig. S3 Panel E).

### HOMA2-IR

Feature importance for HOMA-IR (Fig. 1 Panel F), in the scaled dataset, showed that acelsulfame, an artificial sweetener, was the only significant predictor. Tartronate-hydroxymalonate and methyl-4-hydroxybenzoate-sulfate, were nearly significant in these models. In prediction models with unscaled predictors, MRS—liver fat (βeta 1.13; 95% CI, 1.011 to 1.26) , pyruvate (βeta 2.28; 95% CI, 1.42 to 3.67) and Xanthine (βeta 1.67; 95% CI, 1.047 to 2.67), were the strongest predictors (Supplementary Fig. S3 Panel F).

### Predictors for glucose tolerance test

**Figure 2:**
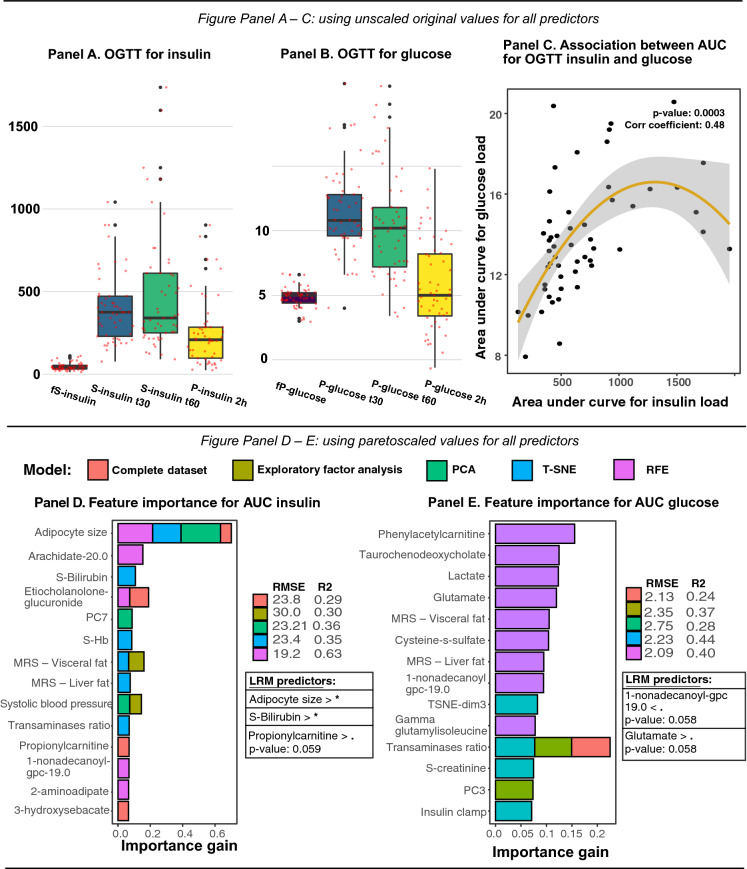
Distribution and area under curve for oral-glucose tolerance test of insulin and glucose, as well as feature importance for constructed variables based on extreme gradient boosting models and linear regression. Panel (**A**)–(**B**) shows distributions for insulin and glucose related OGTT variables that were used to generate AUC variables for insulin and glucose. Panel C shows association between AUC insulin and AUC glucose, along with correlation coefficient. In Panel (**D**)–(**E**), relative importance for predictors generated by extreme gradient boosting models, using pre-processing techniques for metabolomics data to reduce number of predictors in the final model. Model diagnostics (**RMSE**) and validation (**R**^**2**^) are presented next to each prediction model. The most important predictors identified through prediction modeling were included in a linear regression model. Significance level are described as follows: *p-value < 0.05, *p*-values < 0.06. < denotes that lower levels for the predictor was associated with the target variable. > denotes that higher levels for the predictor was associated with the target variable.

Predictors for insulin and glucose metabolism derived by means of oral glucose tolerance test, were amalgamated with a trapezoid formula to describe the area under the curve for OGTT related insulin- and glucose variables. In Fig. 2 Panel A–B displays the distribution of OGTT for insulin and glucose, whilst Fig. 2 Panel C shows the scatter plot for AUC glucose and AUC insulin, along with the correlation for these newly constructed variables.

#### Mean insulin (AUC insulin)

In Fig. 2 Panel D, the prediction model based recursive feature elimination demonstrated superior model diagnostics (R^2^ 0.63). Prediction models with scaled predictors reveled that the most important and statistically significant predictors for mean insulin (AUC insulin) was adipocyte cell size, serum-bilirubin and propionylcarnitine was almost significant (Fig. 2 Panel D). The unscaled models revealed that adipocyte cell size and 1-palmitoyl-2-alpha-linolenoyl-gpc was the most important predictors, however only the last mentioned was significant in the regression model (βeta 1.52; 95% CI, 1.009 to 2.12) were also predictive of mean insulin (AUC insulin) (Supp Fig. S4 Panel B).

#### Mean glycemia (AUC glucose)

In Fig. 2 Panel E, strongest predictors for mean glycemia (AUC glucose) were glutamate and 1.non-adecanoyl-gpc, as compared to the unscaled models, which revealed that trans-urocanate, isovalerylcarnitine, MRS—liver fat and hyocholate, were the most important and significant predictors (Supp Fig. S4 Panel A).

### Adipose tissue morphology

#### MRS—liver fat

**Figure 3:**
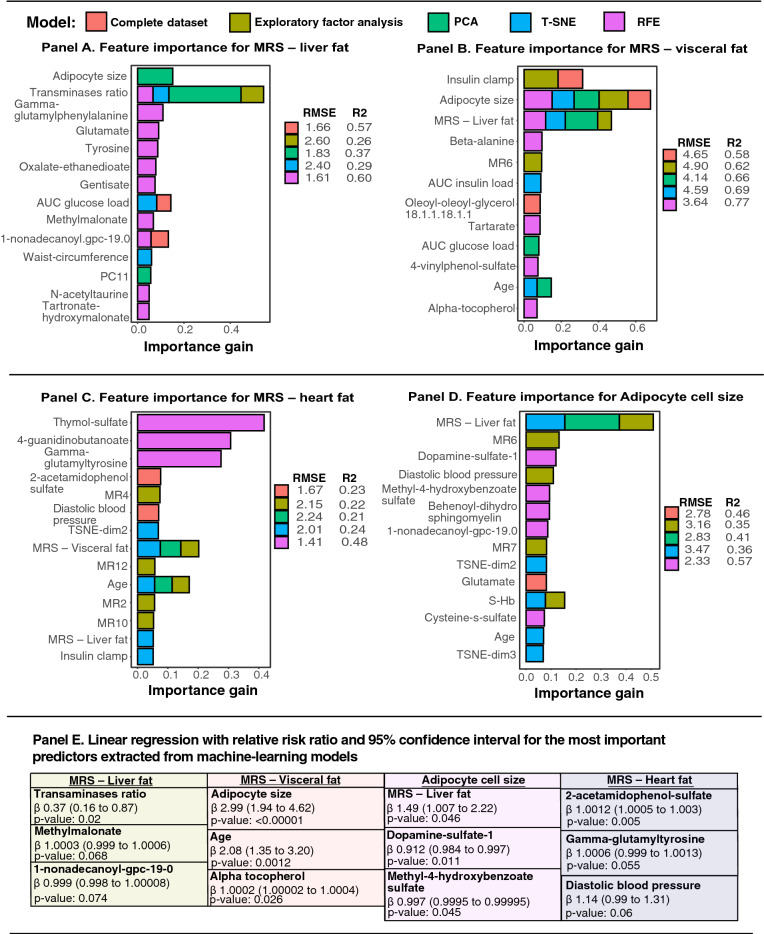
Feature importance for visceral fat and ectopic liver- and heart fat based on extreme gradient boosting models and linear regression for the most important predictors. In Panel (**A**)–(**B**), relative importance for predictors generated by machine learning are presented. Model diagnostics (**RMSE**) and validation (**R**^**2**^) are presented next to each prediction model. In Panel (**E**), the most important predictors identified through prediction modeling were included in linear regression model.

In Fig. 3 Panel A, prediction models for liver fat demonstrated relatively low R^2^ score but comparable RMSE values between models. Linear regression based on machine learning models for the scaled dataset, suggests that liver transaminases, methylmalonate and 1-nonadecanoyl-gpc, were statistically significant (Fig. 3 Panel E). Linear regression based on the unscaled dataset, revealed that adipocyte size (βeta 5.89; 95% CI, 0.74 to 46.7), transaminases ratio (βeta 0.46; 95% CI, 0.23 to 0.91), 1-nonadecanoyl-gpc-19.0 (βeta 0.24; 95% CI, 1.10 to 0.57) and gamma-glutamylphenalylalanine (βeta 10.5; 95% CI, 2.60 to 43.01), were statistically significant (Supp Fig. S5 Panel A).

#### MRS—visceral fat

In Fig. 3 Panel B, virtually all data pre-processing techniques demonstrated robust model diagnostics with high R^2^ value and relatively comparable RMSE value. Adipocyte cell size, age and alpha-tocopherol, were prevalent in most gradient boosting models and statistically significant in linear regression model (Fig. 3 Panel E). As compared to the unscaled models, were adipocyte cell size, age and systolic blood pressure were important in the prediction models and statistically significant in linear regression (Supp Fig. S5 Panel B and Panel E).

#### MRS—cardiac lipids

For cardiac lipids, prediction models had low R^2^ value, in both the unscaled and scaled models. In Fig. 3 Panel C, visceral fat and age demonstrated high feature importance in three prediction models, respectively. However, linear regression, based on scaled prediction models, showed that age and 2-acetamidophenol-sulfate, gamma-glutamyltyrosine and diastolic blood pressure, were statistically significant. Results from the unscaled dataset revealed relatively similar feature importance as the scaled dataset (Supp Fig. S5 Panel C), however linear regression showed that MRS—visceral fat was nearly statistically significant (βeta 2.27; 95% CI, 0.96 to 5.40) (Supp Fig. S5 Panel E).

#### Subcutaneous adipocyte cell size

In Fig. 3 Panel D, liver fat according to magnetic resonance spectroscopy, dopamine sulfate-1 and methyl-4-hydroxybenzoate sulfate, were the most important predictors for adipocyte cell size and statistically significant in the linear regression model. In supplementary Fig. S5 Panel D (unscaled models), prediction models identified methyl-4-hydroxybenzoate sulfate and MRS—liver fat as important predictors, whereas linear regression showed that MRS—liver fat, dopamine-sulfate 1 and sphingomyelin d18.1, were statistically significant.

### Clustering analyses

**Figure 4:**
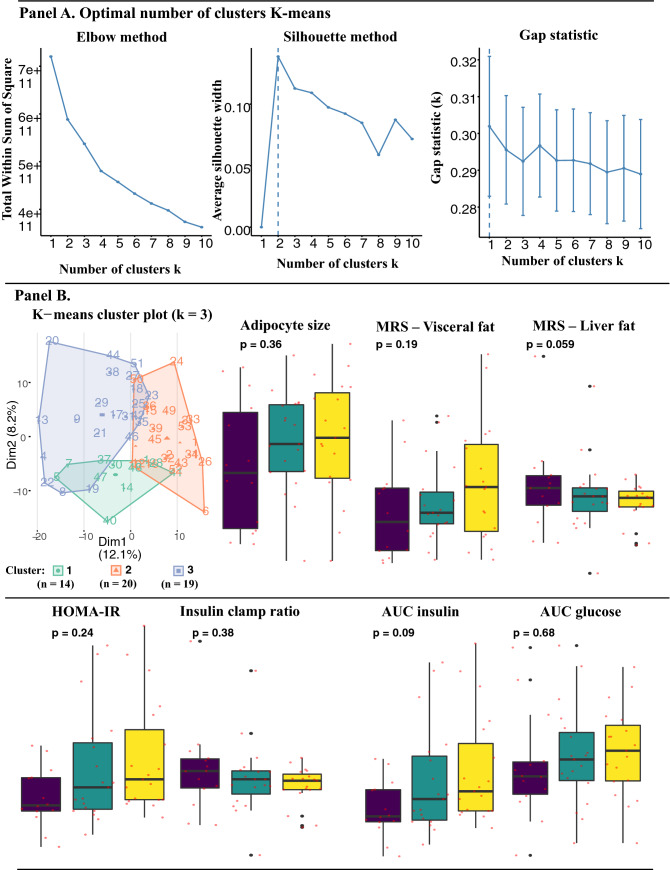
Cluster analysis including validation methods, distribution measures with ANOVA for adipocyte size, magnetic spectroscopy for visceral and ectopic liver fat, and insulin- and glucometabolic predictors. K-means clustering with validation methods revealed that 2 to 3 unique clusters were optimal for this dataset. Thereafter, mean values for predictors of interest in this study was examined and ANOVA was performed to identify significant differences in mean values.

K-means clustering was used to distinguish study participants with unique metabolic phenotypes. We experimented with two to four clusters as the optimal number of cluster groups. Results from K-means clustering (k = 3) are presented in Fig. 4, along with the three different methods that were used to identify optimal number of clusters. Predictors were scaled prior to clustering, similar to our approach for dimensionality reduction. Predictors for study participants belonging to unique clusters were thereafter transformed backwards to unscaled original values and characteristics between the groups, were analyzed with ANOVA. In Fig. 4 Panel B, mean insulin (AUC insulin) and liver fat, were the only predictors that were nearly statistically significantly between the cluster groups. Baseline characteristics for cluster groups are presented in Table 1.

**Figure 5:**
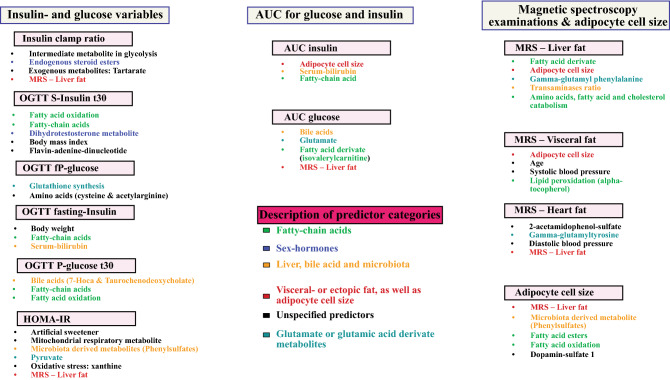
Overview of important predictors for glucose- and insulin metabolism, as well as, radiological examinations and adipocyte cell size. Relative importance for predictors was ranked according to highest relative contribution in machine learning models and thereafter with linear regression models. The most important and significant predictors for each model is presented in this summary figure.

In Fig. 5, a summarizing figure is presented to describe specific predictors or biological processes that were identified through the machine learning models and linear regression analyses.

### Ancillary analyses

In supplementary Fig. S1 Panel A, cumulative variance for the first 20 principal components is presented. In Supp Fig. S1 Panel B–C, parallel analysis to assess optimal number of exploratory factors and hierarchical clustering are presented, respectively. Supplementary Fig. S2 Panel A–B, presents density plots for outcome variables and an ancillary analysis to identify metabolites with strongest correlation to age.

## Discussion

The data obtained and analyzed in this study as well as previously published articles on the 53 subjects is to our knowledge unique in its extensive nature, combining clinical, biochemical, radiological and untargeted serum metabolomics data for comprehensive phenotypic metabolic characterization, as well as enhancing our knowledge of adipocyte biology and insulin- and glucose metabolism. In this study, our primary objective was to examine markers of insulin and glucose metabolism, whilst considering complete untargeted serum metabolomics. Moreover, we presented k-means and hierarchical cluster analyses in an attempt to identify unique metabolic phenotypes, considering our high-dimensional dataset. Predictive machine learning models were constructed in a stepwise fashion with additional pre-processing techniques to reduce number of predictors for each outcome. Our approach demonstrated that relative importance of serum metabolites outperformed traditional clinical and biochemical variables for most endpoints.

Predictive machine learning models based on oral glucose- and insulin tolerance tests, highlighted several metabolites as the most important predictors for glucose and insulin metabolism. Fasting glucose was associated with a known biomarker of obesity, namely cysteine-s-sulphate, which is involved in intracellular insulin action^13^ and n-acetylgarginine, which has been suggested to modulate glucose homeostasis, insulin sensitivity and promote lipolysis, through arginine-nitric oxide modulation of intracellular AMPK and PI3K^14^. In addition, cysteine is involved in gluthathione synthesis, which is known for its relation to beta-cell dysfunction.

Fasting insulin was predicted by body weight, serum-bilirubin and propionylcarnitine. Increased relative importance of propionylcarnitine, a fatty ester lipid molecule, indicates that dysregulated fatty acid metabolism and lipid metabolism in the beta-oxidation of long-chain fatty acids might cause lipid accumulation in tissues, supporting the role as an important metabolite for fasting insulin levels. Carnitine is essential for cellular energy since it transports long-chain fatty acids into the mitochondria for beta-oxidation, as well as transporting toxic compounds out of this cellular organelle to prevent their accumulation. Body weight also demonstrated high relative importance in both scaled and unscaled models, while serum bilirubin was nearly statistically significant.

Glucose levels at 30 min were predicted by 7-Hoca, microbiota derived metabolites, as well as, fatty-chain acids and the metabolite eugenol sulfate, which has been shown to lower blood glucose and blood lipids, as well as lower markers of inflammation^15^. According to animal models, eugenol facilitates insulin sensitivity and stimulates glucose uptake via skeletal muscle tissue and activation of the GLUT4-AMPK signaling pathway.

Both insulin clamp and HOMA-IR, were predicted by metabolites involved in beta-oxidation of fatty acids and biodegradation of triacylglycerol. Tartrate is considered a xenobiotic metabolite that is related to BMI, insulin resistance and adiponectin, while 3-phosphoglycerate is a significant intermediate in glycolysis as well as a non-ATP product of PGK1, which is critical for constructing serine and secreting insulin^16^. According to unscaled predictions models, important predictors included medium chain fatty acid, liver fat according to magnetic resonance spectroscopy, pyruvate and xanthine. Increasing serum levels of xanthine and xanthine oxidoreductase (XOR) has previously shown to be associated with greater production of reactive oxygen species, endothelial dysfunction, body mass index, fasting plasma insulin and insulin resistance. According to the scaled models, the artificial sweetener acelsulfame and methyl-4-hydroxybenzoate-sulfate, as well as, tartronate-hydroxymalonate, which is involved in fatty acid biosynthesis and mitochondrial energy production, proved to be important predictors for HOMA2-IR. Acelsulfame has previously been associated with increasing BMI and glucose intolerance.

Information derived from OGTT was used to calculate an area under curve value (AUC) for both glucose and insulin measures. These newly constructed endpoint variables were associated with several examined metabolites. In scaled prediction models, nonadecanoyl-gpc and glutamate, were almost statistically significant. According to unscaled prediction models, AUC for glucose was associated with bile acid metabolites, fatty acid esters (valerylcarnitine) and liver fat according to magnetic spectroscopy.

AUC insulin was predicted by subcutaneous adipocyte size as well as a metabolite of sphingolipid metabolism, a compound involved both in intracellular signaling and cell membrane turnover, as well as serum-bilirubin. Sphingolipids have previously been shown to be associated with insulin resistance, possibly via downstream insulin signaling alterations^6^. In addition to this, the scaled prediction models identified serum-bilirubin and propionylcarnitine, as important predictors for AUC insulin. Adipocyte hypertrophy has been extensively studied as a mediator in the development of insulin resistance and hyperinsulinemia and our results are in line with previous results^3^.

Finally, subcutaneous adipocyte size was found to be associated with markers of sphingolipid metabolism, dopamine-sulfate 1, liver fat and methyl-4-hydroxybenzoate sulfate, were important predictors for adipocyte cell size. Previous research has suggested a regulatory role for peripheral dopamine-sulfate in adipose tissue.

Clustering analyses identified three unique phenotypic groups, where levels of insulin resistance, defined by insulin clamps, differed significantly between the groups. At a tendency level, amount of visceral liver fat also differed but failed to reach statistical significance. We found several markers of amino acid metabolism that predict visceral adipose tissue, a finding that is in line with previous research as amino acid metabolites have been shown to predict insulin resistance^17^. We also found a bile acid metabolite, as well as a glycolysis metabolite to predict visceral liver fat, two cellular processes we have mentioned previously to be associated with insulin resistance.

In our previous research, we observed that in these subjects both visceral and subcutaneous fat area by MRS evaluation were predicted by metabolites of fatty acid oxidation. Lipid oxidation metabolites also predicted liver lipid accumulation, and cardiac lipid storage was predicted by a metabolite of branched chain amino acid (BCAA) turnover^9^. BCAA have previously been linked to IGT and overt type 2 diabetes and our findings are in line with these results^6,18^. Our findings in this study are thus an addition to previous findings. Ectopic lipid accumulation in liver was predicted by amount of subcutaneous adipocyte cell size, liver transaminases, methylmalonate, lipid metabolites and gamma-glutamylphenylalanice. According to scaled models, predictors for visceral fat were subcutaneous adipocyte cell size, ectopic liver fat and insulin clamp. However, linear regression shows that only adipocyte cell size, age and alpha-tocopherol, were associated with visceral fat. Our data are not able to distinguish whether visceral fat accumulation precedes ectopic fat storage in the liver. In general, repeated cross-validation for the machine learning model for ectopic adipose tissue surrounding the heart tissue was poor, nevertheless age, diastolic blood pressure, 2-acetamidophenol-sulfate, gamma-glutamyltyrosine and visceral fat, were the best predictors.

A major strength of this study is the extensive examination of subjects using clinical and biochemical variables, imaging data and untargeted metabolomics. Some limitations of our study should be considered. The relatively small number of subjects included in our study complicates our ability to cross-validate and generalize our machine learning models. Validation models on test dataset were impracticable in some cases due to size of the cohort. We believe that a trade-off between a lesser regression-mean squared error (RMSE) value and R^2^ is satisfactory in this dataset to signify the superior model for each endpoint.

## Conclusion

We identified several biomarkers associated with markers of dysfunction of adipose tissue and its morphology and insulin and glucose metabolism using a multi-modal machine learning approach.

## Supplementary Information

Supplementary Information 1.
