# Supplementary material for: Identification of markers that distinguish adipose tissue and glucose and insulin metabolism using a multi-modal machine learning approach
Source: Sci Rep. 2021 Aug 23;11:17050. doi: 10.1038/s41598-021-95688-y (PMC8382765; doi:10.1038/s41598-021-95688-y)
Supplement: Supplementary file 1 — Supplementary Information 1. [file 41598_2021_95688_MOESM1_ESM.pdf]

Identification of Markers that Distinguish Adipose Tissue and Glucose and Insulin Metabolism using a Multi-Modal Machine Learning Approach

Supplementary appendix

Supplementary Figure S1: Dimensionality reduction and hierarchical clustering

Supplementary Figure S1. Examination of dimensionality reduction techniques and hierarchical clustering

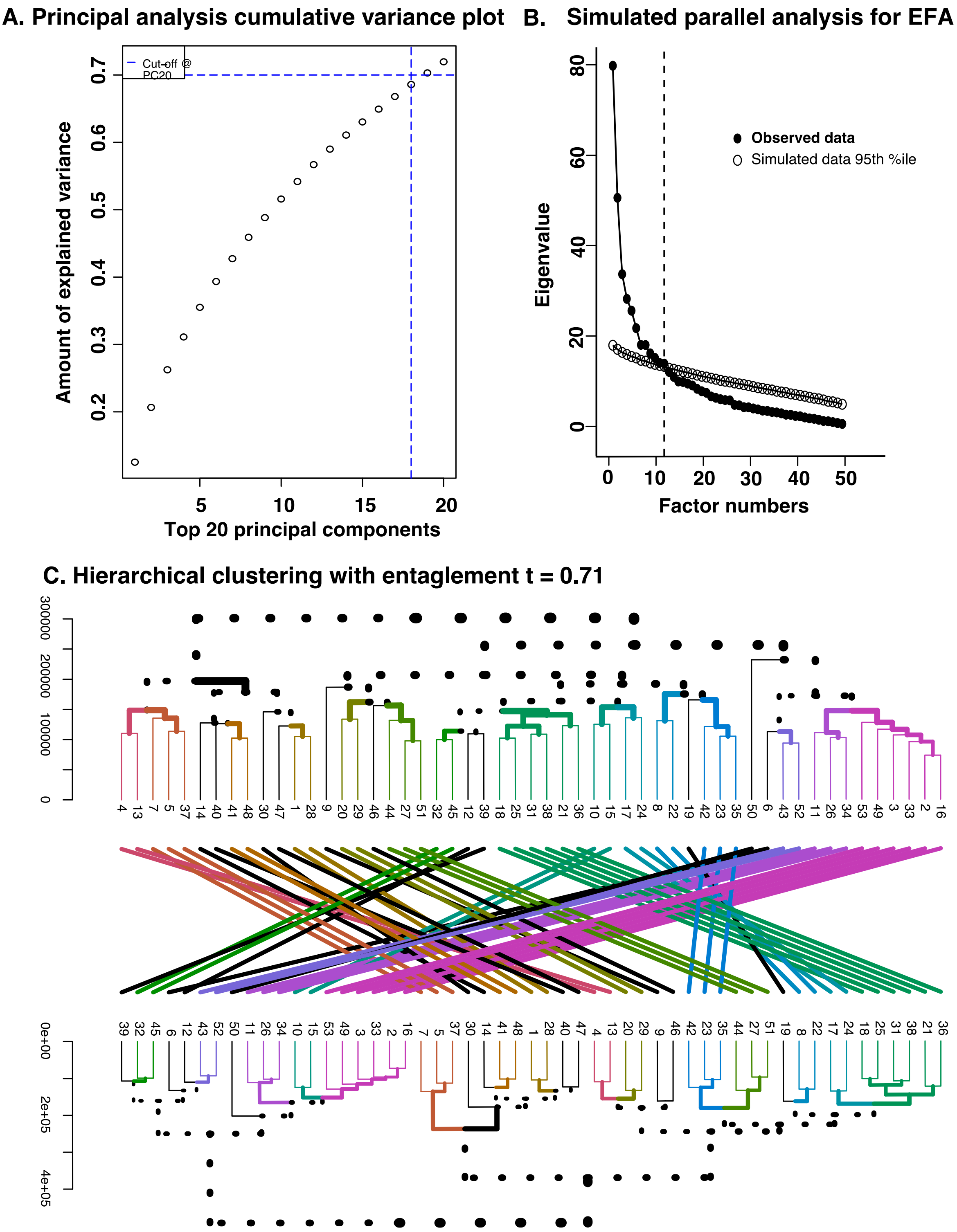

Figure legend: Panel A – B presented cumulative variance for the first 20 principal components and parallel analysis to assess optimal number of factors for metabolites. Panel C presents hierarchical clustering of all study participants.

Supplementary Figure S2: Distribution of variables and correlation between increasing age and metabolites

Supplementary Figure S2: Distribution for variables of interest and created AUC variables for insulin and glucose. Correlationplot is presented for age and metabolites in the dataset.

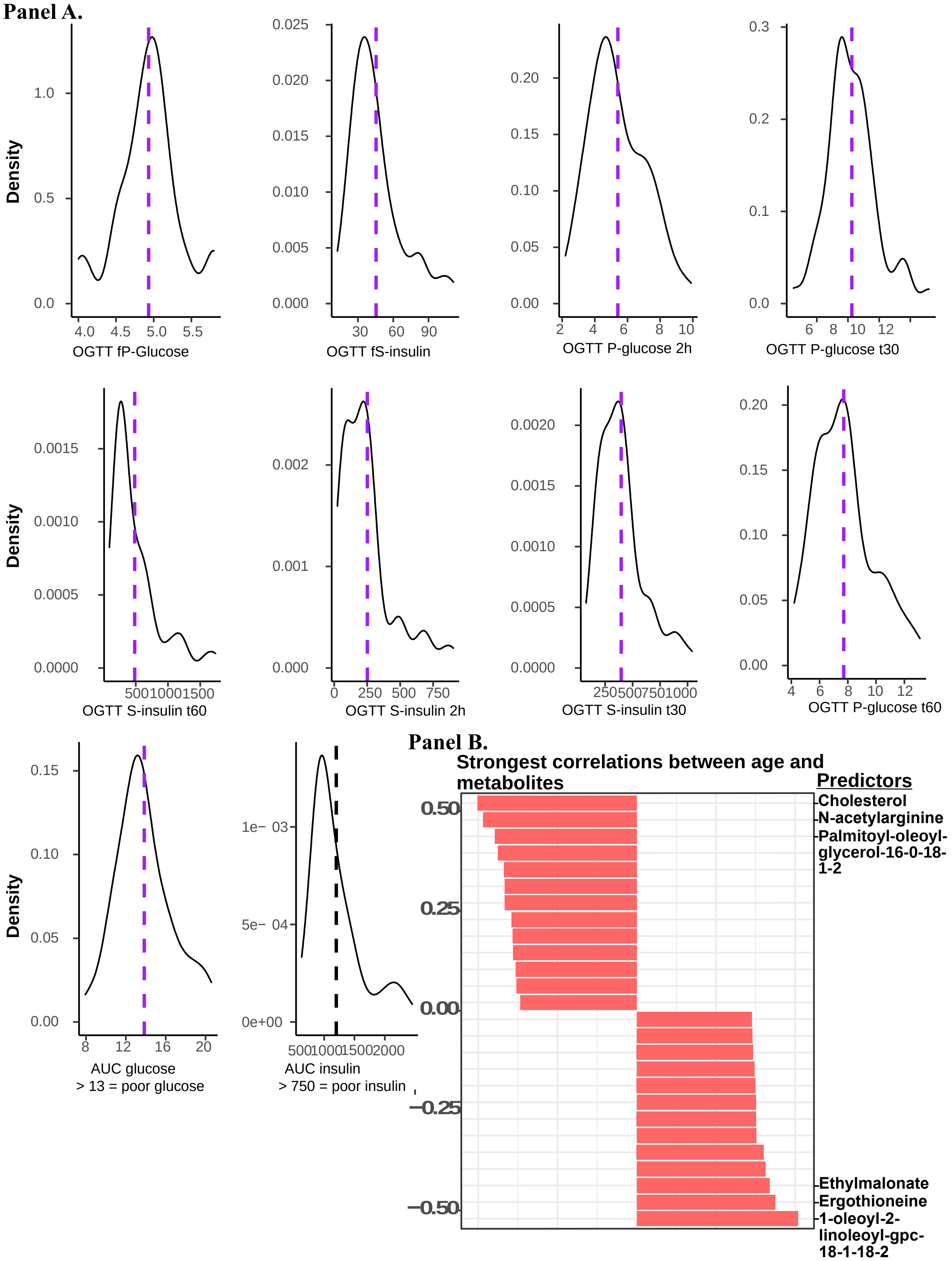

Figure legend: In Panel A, distribution for variables of interest and created AUC variables for insulin and glucose. In Panel B, correlationplot is presented for age and metabolites in the dataset.

Supplementary Figure S3: Feature importance for insulin and glucose predictors (unscaled data)

Supplementary Figure S3. Feature importance for insulin- and glucometabolic markers based on extreme gradient boosting models and linear regression for the most important predictors.

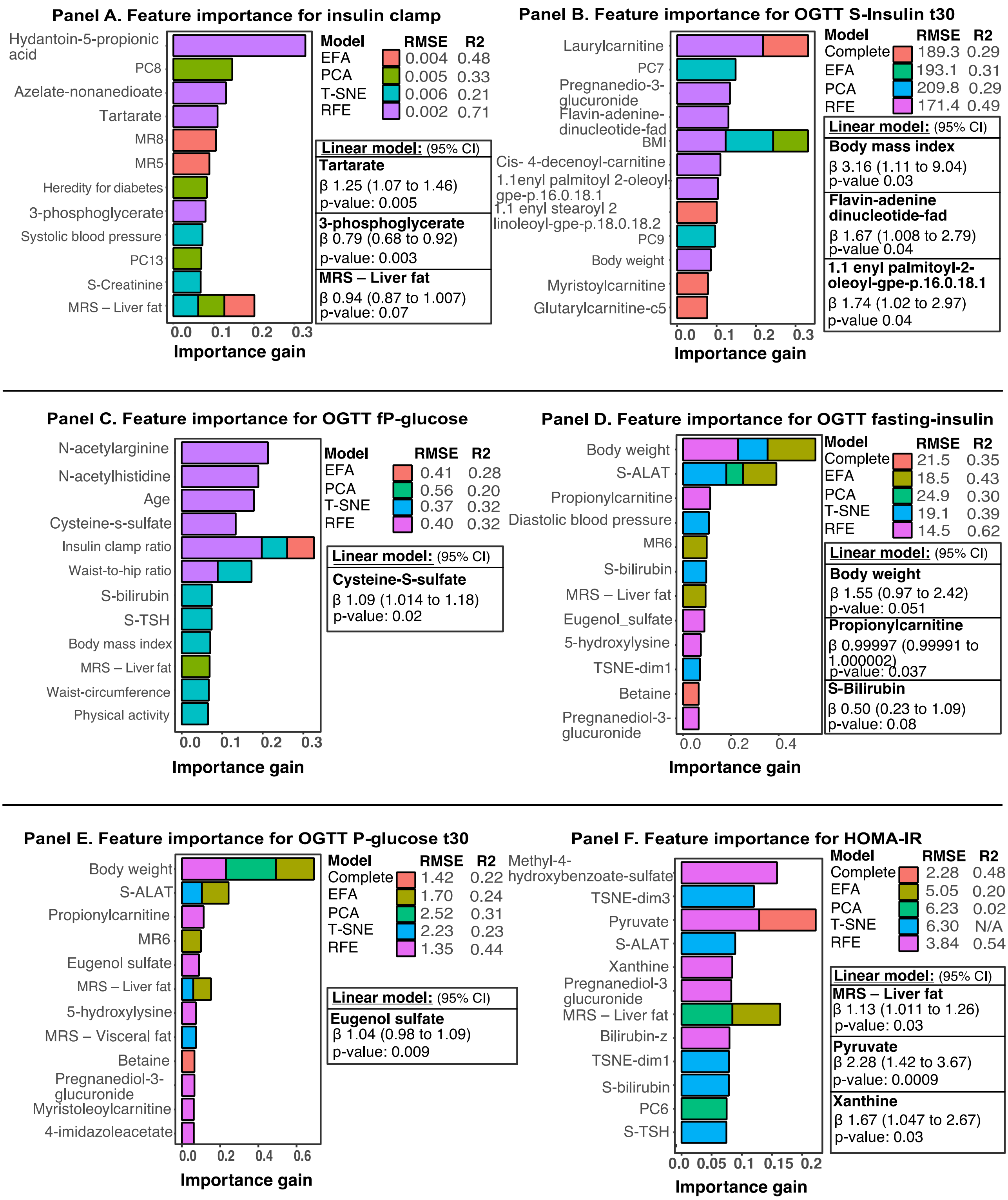

Figure legend: Machine learning models for glucose and insulin related variables with linear regression for the most important predictors for each outcome. Prediction models were based on unscaled predictors and regression models present unstandardized beta-coefficients.

Supplementary Figure S4: Feature importance for AUC glucose and insulin levels (unscaled data)

Supplementary Figure S4. Distribution and area under curve for oral-glucose tolerance test of insulin and glucose, as well as feature importance for constructed variables based on extreme gradient boosting models and linear regression, using unscaled predictors.

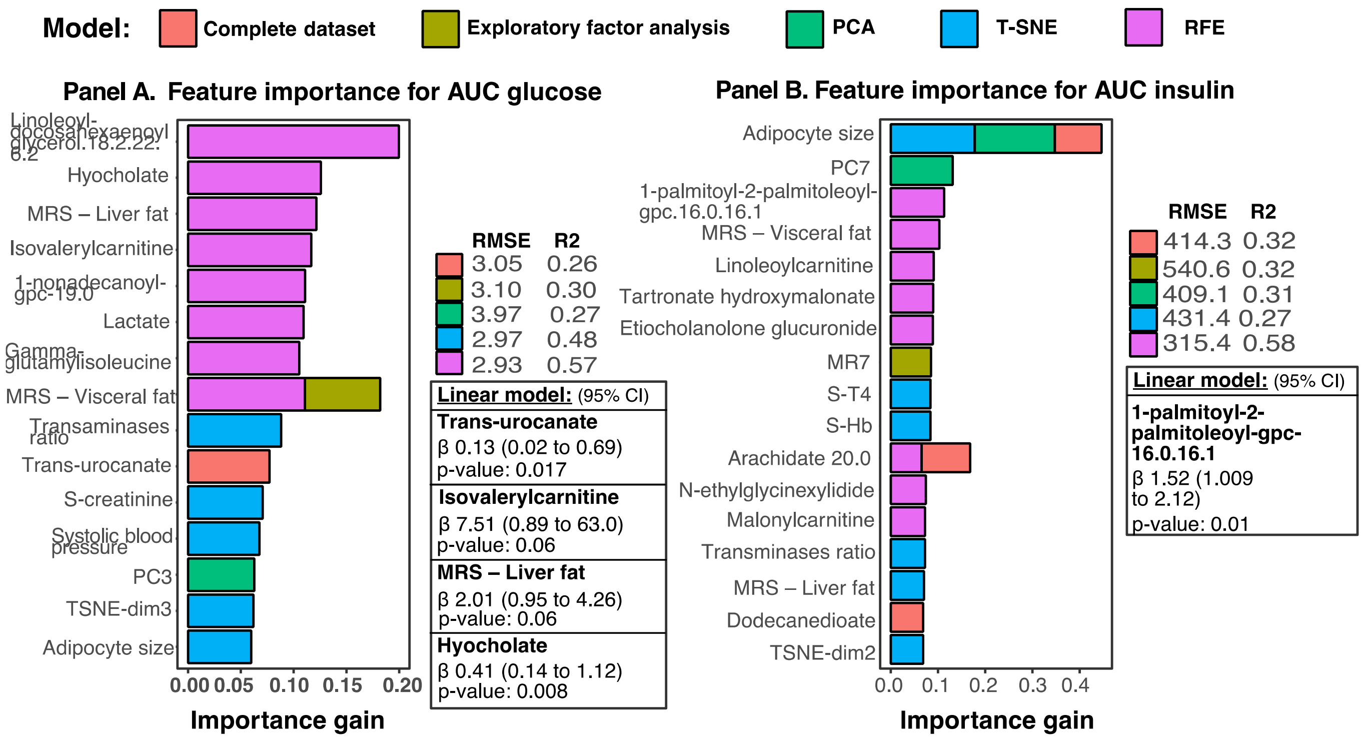

Figure legend: Panel A–B shows relative importance for predictors generated by extreme gradient boosting models, using pre-processing techniques for metabolomics data to reduce number of predictors in the final model. Model diagnostics (**RMSE)** and validation (**R^2**) are presented next to each prediction model. The most important predictors identified through prediction modeling were included in a linear regression model.
Significance level are described as follows: * p-value < 0.05, ** p-value < 0.005, *** p-value < 0.0005 **.** p-values < 0.075
Prediction models were created using unscaled predictors and beta-coefficients are unstandardized.

Supplementary Figure S5: Feature importance for adipocyte fat depots and cell size (unscaled data)

Supplementary Figure S5. Feature importance for visceral fat and ectopic liver- and heart fat based on extreme gradient boosting models and linear regression for the most important predictor.

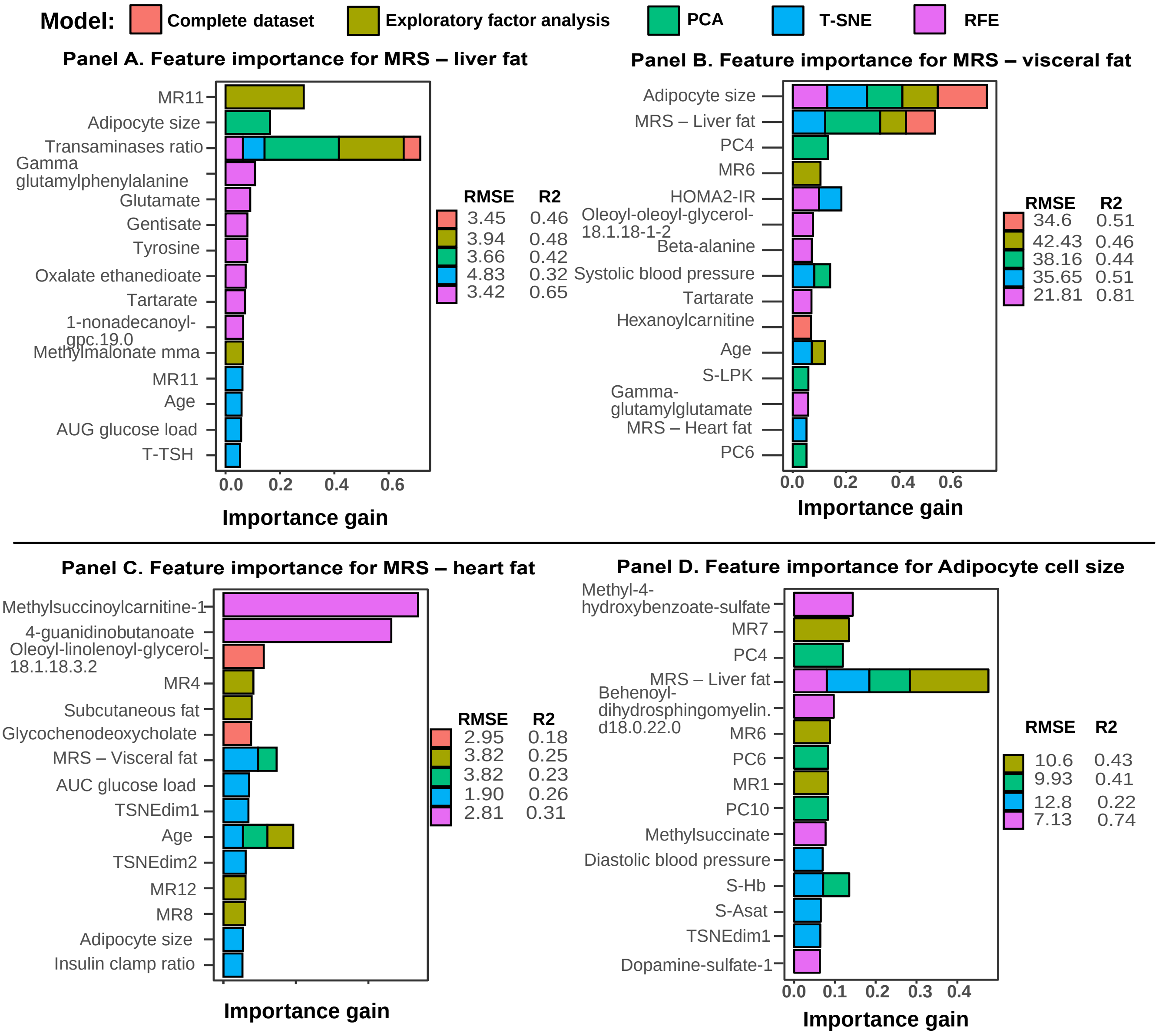

Panel E. Linear regression with relative risk ratio and 95% confidence interval for the most important predictors extracted from machine-learning models

MRS – Liver fat	MRS – Visceral fat	Adipocyte cell size
**Adipocyte size**<br>β 5.89 (0.74 to 46.7)<br>p-value: 0.09	**Adipocyte size**<br>β 18.1 (6.32 to 51.6)<br>p-value: <0.0001	**MRS – Liver fat**<br>β 1.06 (1.03 to 1.09)<br>p-value: <0.0001
**Transaminases ratio**<br>β 0.46 (0.23 to 0.91)<br>p-value: 0.027	**Age**<br>β 2.00 (1.20 to 3.37)<br>p-value: 0.01	**Dopamin-sulfate 1**<br>β 0.92 (0.86 to 0.99)<br>p-value: 0.04
**1-nonadecanoyl-gpc-19.0**<br>β 0.24 (0.10 to 0.57)<br>p-value: 0.001	**Systolic blood pressure**<br>β 2.83 (1.08 to 7.45)<br>p-value: 0.03	**Sphingomyelin d18.1.1 d18.2.18.0**<br>β 1.22 (1.06 to 1.41)<br>p-value: 0.005
**Gamma-glutamylphenylalanine**<br>β 10.5 (2.60 to 43.01)<br>p-value: 0.001	**MRS – Heart fat**<br>**MRS – Visceral fat**<br>β 2.27 (0.96 to 5.40)<br>p-value: 0.060	

Figure legend: Machine learning models for glucose and insulin related variables with linear regression for the most important predictors for each outcome. Prediction models were based on unscaled predictors.

Supplementary Figure S6: Graphical illustration of machine learning model building, validation and performance

Supplementary Figure S6. Graphical illustration of machine learning model building, validation and performance processes

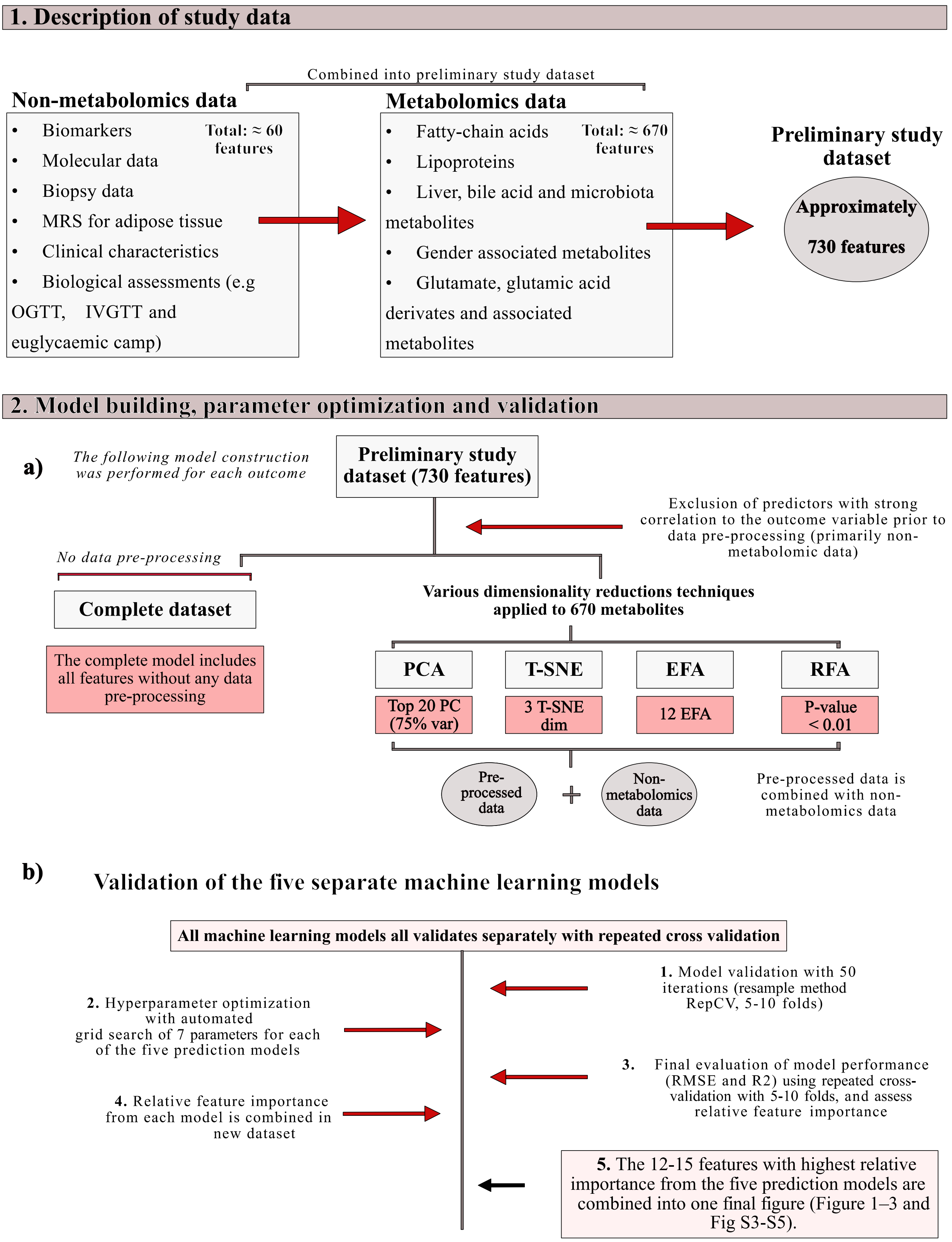

Legend: The figure above demonstrates model construction, hyperparameter optimization and model validation for the prediction models.
